# A Set of Novel Microsatellite Markers Developed for *Luculia yunnanensis* (Rubiaceae), an Endangered Plant Endemic to Yunnan, China

**DOI:** 10.3390/ijms13010534

**Published:** 2012-01-04

**Authors:** Hong Ma, Lan Wang, Youming Wan, Hongzhe Li, Zhenghong Li, Xiuxian Liu, Ning Liang, Wenjuan Li

**Affiliations:** 1 Research Institute of Resource Insects, Chinese Academy of Forestry, Kunming 650224, China; E-Mails: mahongmh@gmail.com (H.M.); wanyouming@126.com (Y.W.); lxx205@126.com (X.L.); ln417@126.com (N.L.); hanbing2587cn@yahoo.com.cn (W.L.); 2 Yunnan Reascend Tobacco Technology (Group) Co., Ltd., Kunming 650106, China; E-Mail: lwang0124@gmail.com; 3 Faculty of Traditional Chinese Pharmacy, Yunnan University of Traditional Chinese Medicine, Kunming 650500, China; E-Mail: lihongzheyn@163.com

**Keywords:** genetic diversity, *Luculia yunnanensis*, microsatellite marker, polymorphism, population structure, Rubiaceae

## Abstract

The genus *Luculia* Sweet contains about five species of small trees or shrubs and is a member of the family Rubiaceae (tribe Cinchoneae)*. Luculia yunnanensis* is an endangered ornamental shrub endemic to southwest China. Only two natural populations of *L. yunnanensis* exist in the wild according to our field investigation. It can be inferred that *L. yunnanensis* is facing a very high risk of extinction in the wild and an urgent conservation strategy is required. By using a modified biotin-sterptavidin capture method, 24 primer sets were identified in two wild populations. Of these primers, 11 displayed polymorphisms and 13 were monomorphic. The number of alleles per locus ranged from two to four, values for observed and expected heterozygosities ranged from 0.000 to 0.833 and from 0.431 to 0.771, with averages of 0.389 and 0.614, respectively. These markers will be useful for further investigation of conservation of resources, selecting parental types in cross-breeding, evolution of this species at the molecular level and related research in *Luculia* species.

## 1. Introduction

The genus *Luculia* Sweet contains about five species of small trees or shrubs and is a member of the family Rubiaceae (tribe Cinchoneae) [1]. It can be easily recognized by its beautiful pink, sweetly fragrant and long-term blooming time flowers, and therefore has been horticulturally introduced into various regions and botanical gardens around the world. Three *Luculia* species exist in China; among them *L. yunnanensis* Hu is the unique one which is endemic to Yunnan Province, naturally confined to forests or thickets on limestone mountains, open slopes and secondary shrubby woodland at an altitude of between 1200 and 3200 m [2]. It is a typical distylous species with reciprocally placed stigma and anthers in each floral morph [3]. Nevertheless, our continued field investigations showed that it is now difficult to find wild populations as appropriate habitats have been fragmented due to human activities.

Considering its high ornamental value, endemism and endangered status, we developed 24 microsatellite markers from *L. yunnanensis* to obtain its genetic information for further studies on conservation of resources, selecting parental types in cross-breeding, as well as evolution of this species at the molecular level.

## 2. Results and Discussion

A total of 317 putative SSR-positive clones were captured, among these 165 clones (52%) were found to contain simple sequence repeats (SSR). Finally, 48 sequences contained SSR loci were selected for primer design. Twenty-four microsatellite loci successfully amplified in *L. yunnanensis* for 48 microsatellite loci and 11 of them were polymorphic amplification, other 13 microsatellite loci were monomorphic by the result of polyacrylamide gel (Table 1).

The number of alleles ranged from two to four in 24 individuals of the species sampled from the two natural populations. Values for *H*_O_ and *H*_E_ ranged from 0.000 to 0.833 and from 0.431 to 0.771, with averages of 0.389 and 0.614, respectively (Table 2).

These microsatellite markers developed in our study will be a useful tool for further studies of conservation genetics, and will help us understand the genetic structure of *L. yunnanensis*, so as to make effective conservation strategy for this endangered plant.

## 3. Experimental Section

Genomic DNA samples of *L. yunnanensis* were extracted from silica-gel-dried leaves using a modified CTAB methodology [4]. The extracted DNA was dissolved in 30 μL TE buffer. A microsatellite enriched library was then conducted using a modified biotin-streptavidin capture method [5]. Total genomic DNA (approximate 250–400 ng) was completely digested with 2.5 U of MseI restriction enzyme (New England Biolabs, Beverly, MA, USA), and then ligated to an MseI AFLP adaptor (5′-TAC TCA GGA CTC AT-3′/5′-GAC GAT GAG TCC TGA G-3′) using T4 DNA ligase (New England Biolabs, Beverly, MA, USA). The digested-ligated fragments were diluted in a ratio of 1:10, and 5 μL of them were used amplification reaction with adaptor-specific primers (5′-GAT GAG TCC TGA GTA AN-3′/5′-TTA CTC AGG ACT CAT CN-3′). The amplified DNA fragments (200–800 bp) were enriched by magnetic bead selection with a 5-biotinylated [(AG)_15_, (AAG)_10_ and (AC)_15_] probe, respectively [6]. The Recovered DNA fragments were reamplified with *Mse*I-N primers. The purified PCR products using EZNA Gel Extraction Kit (Omega Bio-Tek, Guangzhou, China), were ligated into pMD18-T vector (Takara, Dalian, Liaoning, China), and then transformed into DH5*a* competent cells (Tiangen, Beijing, China). The positive clones were tested using vector primers T3/T7 and primer (AC)_10_/(AG)_10_/(AAG)_7_ respectively. All these PCR reactions had the same conditions: 95 °C for 3 min followed by 32 cycles at 94 °C for 45 s, 52 °C for 1 min, 72 °C for 1 min, and a final extension step at 72 °C for 10 min. The positive clones were captured for sequencing with an ABI PRISM 3730XL DNA sequencer (Applied Biosystems, Foster City, CA, USA). Sequences contained simple sequence repeat and enough flanking regions were selected for primer design using Primer Premier 5.0 program [7].

The designed Primer pairs were tested in 24 wild individuals of *L. yunnanensis* from two natural populations collected in Southwest China: population 1 (Lushui County, 26°29'N, 98°50'E, 1772a.s.l.) and population 2 (Fugong County, 25°58'N, 98°47'E, 1884a.s.l.). Herbarium voucher deposited in Kunming Institute of Botany, Chinese Academy of Sciences. The PCR amplification was carried out in a total volume of 20 μL reaction containing 10 μL 2 × Taq PCR MasterMix (Tiangen; 0.1 U Taq Polymerase/μL, 0.5 mM dNTP each, 20 mM Tris-HCl (Ph 8.3), 100 mM KCl, 3 mM MgCl_2_), 0.5 μL of each primer and 50–100 ng Genomic DNA. PCR amplifications were conducted under the following conditions: 95 °C for 5 min followed by 32–35 cycles at 94 °C for 45 s, at the annealing temperature for each specific primer (optimized for each locus, Table 1) for 45 s, 72 °C for 1 min, and a final extension step at 72 °C for 10 min. PCR products were separated on 8% polyacrylamide denaturing gel using a 20 bp ladder molecular size standard by silver staining.

The data was analyzed by GENEPOP 4.0 [8], which included test of observed heterozygosity (*H_O_*) and expected heterozygosity (*H_E_*) for the 11 polymorphic microsatellite loci.

## 4. Conclusions

In summary, 24 microsatellite markers have been specifically developed for *L. yunnanensis* in this study. These markers will facilitate further studies on the population genetics of *L. yunnanensis* and its allied species. They are also expected to be useful for parental selections in controlled hybridization breeding programs and enable us to protect sources of germplasm through genetic diversification via *ex situ* nurseries and reintroduction programs.

## Figures and Tables

**Table 1:** Characteristics of 24 microsatellite loci successfully amplified in *Luculia yunnanensis*.

Locus	Primer sequence (5′-3′)	Repeat motif	Size (bp)	Ta (°C)	GenBank Accession No.
MH 01 *	F: GCACTGGCTTAGTTCTTT R: TGTTTGTCTCAGGGGTAT	(CT)12(CCCT)3(CT)4	213	60	JN871296
MH 02 *	F: AAACGAGCAGAAACTGGA R: GCATTGTCCAACCCTTTA	(TG)11(AG)9	153	60	JN871297
MH 03 *	F: CAACTTCCCAAATTCTGC R: TACCCAACACTTTCTCACCA	(CT)16	322	61	JN871298
MH 04 *	F: TTTCATTCCGTAACCTT R: GCCAAAAACTTGTATAATTC	(CT)33	198	57	JN871299
MH 05 *	F: TCGTACAATTTTGTTGCTCT R: CATTCCTCAATAAACCCATA	(TG)12	130	61	JN871300
MH 06 *	F: CTCTATCTGTCGCATTCTTC R: TCTAGTAATACTATCCTCCACC	(TC)19	201	61	JN871301
MH 07 *	F: ACCTCGGATAGAAAACAGC R: GTCGTATATAAAGTAAAGCATTGCC	(GA)4(CA)6(GA)14	284	57	JN871302
MH 08 *	F: TGAACTCCCCAGTCCCCACT R: TCGGTCCACAATCATCACAAAA	(AG)22	187	62	JN871303
MH 09 *	F: TGGTCATCAACTATTTTCCCTC R: ATGTATTCATTCAGGTATAGAGAGA	(TC)14	153	57	JN871304
MH 10 *	F: GTCCTCCATAGCCACAAA R: TACTATAACTGATTTTCAAATTTCC	(GAA)13	130	59	JN871305
MH 11 *	F: GTCCTAGTGTACTTACCCATCA R: GACAGCAATGCTCCGACT	(CA)14	192	61	JN871306
MH 12	F: CAATTCTTGAGCCACTTT R: GCTTCTGCCAATGTTTAG	(GA)13	208	56	JN871307
MH 13	F: AAGAAGGTGAAGGAGGCT R: AGATGGTCCCAATGTAGC	(TC)12(GT)7(CT)4	262	49	JN871308
MH 14	F: GGGTTCAAGGCTCAGGAC R: CATCCCATTGCCAACATC	(GA)15	221	49	JN871309
MH 15	F: TATGCTTGGGCAGGAATG R: AACGCTGTGCTATCACTGTCTT	(GA)21	201	54	JN871310
MH 16	F: TTTTAGTAGGGTTCACCG R: CCCACTCCTGGATGTTTG	(CT)32	192	54	JN871311
MH 17	F: ATTCTGTATCTGGCTCAC R: GTCTTTCAAGCATAAAGA	(CT)14(CA)11	279	55	JN871312
MH 18	F: ACGAGCAGAAACTGGAAA R: GCATTGTCCAACCCTTTA	(GT)11(GA)9	153	58	JN871313
MH 19	F: TCTACTTCGGTTCAGGGTT R: GCTTCCGCTGTAATGGTT	(TC)25(AC)5	283	61	JN871314
MH 20	F: ATGACTCCACATAGCAAACA R: CTGGGCTGATCCTAAACA	(AG)20	233	63	JN871315
MH 21	F: GGGAAATGGTTCTTTATGGT R: TCATCATGGGTTGGGTTAT	(CT)21	142	59	JN871316
MH 22	F: CATCAACTCACGATTGCA R: CGTTATGTTTCTTTTCTCCC	(TA)6(GA)16	236	55	JN871317
MH 23	F: GCAGGTGAGAATGCAAAT R: AAGCAGCATCACAGTTCC	(AG)27(TG)4	157	59	JN871318
MH 24	F: GCTTGTTTTGATGGTTGT R: TAGGCGAATGATGCTTAT	(CA)13(GA)25	424	55	JN871319

* Displayed polymorphisms in *Luculia yunnanensis*; Ta, PCR annealing temperature.

**Table 2:** Results of 11 polymorphic microsatellite loci screening in two populations of *Luculia yunnanensis*.

	Population 1 (*N* = 12)			Population 2 (*N* = 12)		

Locus	*N*_A_	*H*_O_	*H*_E_	*N*_A_	*H*_O_	*H*_E_
MH 01	4	0.833	0.684	3	0.666	0.489
MH 02	2	0.333	0.463	3	0.250	0.539
MH 03	3	0.000	0.652	4	0.000	0.681
MH 04	3	0.250	0.561	3	0.250	0.684
MH 05	4	0.416	0.655	4	0.583	0.771
MH 06	3	0.166	0.681	4	0.500	0.753
MH 07	2	0.333	0.463	5	0.583	0.768
MH 08	3	0.166	0.594	4	0.500	0.699
MH 09	4	0.250	0.587	4	0.583	0.655
MH 10	2	0.250	0.431	2	0.666	0.463
MH 11	3	0.666	0.648	3	0.333	0.594

*N*, population sample size; *N*_A_, number of alleles revealed; *H*_O_, observed heterozygosity; *H*_E_, expected heterozygosity.
